# Patient Perceptions of Artificial Intelligence and Telemedicine in Dermatology: Narrative Review

**DOI:** 10.2196/75454

**Published:** 2025-09-16

**Authors:** Charlotte McRae, Ting Dan Zhang, Leslie Donoghue Seeley, Michael Anderson, Laci Turner, Lauren V Graham

**Affiliations:** 1 Department of Dermatology, University of Alabama at Birmingham Heersink School of Medicine, 510 20th Street South FOT 858, Birmingham, AL, 35294, United States

**Keywords:** digital health, technology, patient-centered care, health care innovation, trust, convergence, artificial intelligence, teledermatology

## Abstract

**Background:**

Artificial intelligence (AI) and telemedicine have significant potential to transform dermatology care delivery, but patient perspectives on these technologies have not been systematically compared.

**Objective:**

This study aimed to examine patient perspectives on AI and telemedicine in dermatology to inform implementation strategies as these technologies increasingly converge in clinical practice.

**Methods:**

A comprehensive literature search was conducted using PubMed, Scopus, and Embase databases between August 2024 and October 2024. We identified 48 papers addressing patient perspectives on AI and telemedicine in dermatology, with none directly comparing patients’ views of both technologies.

**Results:**

Several distinct themes emerged regarding patient perspectives on these technologies: willingness to use, perceived benefits and risks, barriers to implementation, and conditions necessary for successful integration. Findings revealed that patients express hesitancy toward AI-based diagnoses that lack dermatologist involvement, while preferences for teledermatology varied by reason for appointment, age, and previous technology exposure. Patients’ motivations for implementing AI are connected to its potential for quicker diagnoses and improved triage efficiency. At the same time, telemedicine addresses logistical challenges such as reduced travel time and improved appointment availability. Both technologies were perceived to improve accessibility and diagnostic efficiency, though patients expressed concerns about AI’s limited communication abilities and teledermatology’s inability to perform physical examinations. Primary adoption barriers for these modalities included technological limitations and trust concerns, with patients emphasizing the need for dermatologist oversight, transparency, and adequate educational resources for successful integration.

**Conclusions:**

The complementary strengths of AI and teledermatology suggest they could mitigate each other’s limitations when integrated—AI potentially enhancing teledermatology’s diagnostic accuracy, while teledermatology addresses AI’s lack of human connection. By thoroughly examining these perspectives, this review may serve as a guide for the patient-centered integration of technology in the future landscape of accessible dermatologic care.

## Introduction

Artificial intelligence (AI) and telemedicine have the potential to transform health care delivery in dermatology, where they serve distinct yet complementary functions. AI is a branch of computer science that involves the automation of intelligent behavior [1]. Machine learning, a subfield of AI, applies large datasets to identify patterns for diagnosis and predict clinical outcomes in medicine [1]. In dermatology, AI applications include tools that classify dermatological images obtained from outside clinical settings, as well as clinician decision-support systems that analyze images of patient skin concerns at the point of care [1].

Complementing these AI innovations, telemedicine has experienced rapid growth, specifically after the COVID-19 pandemic catalyzed its widespread adoption as an alternative to face-to-face consultations amid continued service demands [2,3]. Teledermatology, the subset of telemedicine specific to dermatology, offers various delivery modalities. These include synchronous approaches, which involve real-time communication between the patient and dermatologist, asynchronous methods where a dermatologist evaluates clinical images at a later time, and hybrid models combining both approaches [4]. Through these avenues, teledermatology enables remote consultative recommendations, prioritization of care through remote triage, and monitoring of chronic conditions [5].

The integration of AI within teledermatology platforms represents a natural progression in dermatologic care delivery [6]. AI algorithms can enhance teledermatology visits by providing real-time image quality assessment, automated prescreening of cases, and diagnostic decision support during online consultations [7,8]. This convergence could benefit underserved populations by combining AI’s diagnostic capabilities with the remote accessibility advantages of telemedicine [9]. While this technological integration enables advanced, location-independent models for the future of accessible dermatologic care [4], both technologies face unique challenges, and their convergence could potentially either amplify or ameliorate the barriers and limitations of each. Thus, considering patient perspectives on each modality is paramount to promoting the responsible use of these technologies in dermatology as remote care platforms evolve into influential components of dermatologic practice [10,11]. By examining patient perspectives on both AI and teledermatology, this review aims to inform implementation strategies that capitalize on synergistic benefits while addressing challenges, serving as a guide for the patient-centered integration of technology in dermatologic care.

## Methods

### Overview

A comprehensive literature search was conducted using the PubMed, Scopus, and Embase databases between August 2024 and October 2024. To identify relevant papers addressing patient perspectives on AI and telemedicine in dermatology, we used the following search terms: ((artificial intelligence) AND (dermatology)) AND (comfort OR perception OR perspective), as well as ((Telemedicine OR Teledermatology) AND (dermatology)) AND (comfort OR perception OR perspective).

The initial search yielded 622 papers, which were imported into Covidence for systematic screening. After removing 236 duplicates, 386 studies underwent independent title and abstract review by three researchers (CM, TDZ, and LDS). Conflicts were resolved through discussion until a consensus was reached. Following the initial screening, 60 papers underwent a full-text review, with a focus on original research and data-supported observations. Ultimately, 38 papers met our inclusion criteria: studies addressing patient perspectives or perceptions of AI and telemedicine in dermatology, published after 2009, in English with full text available, and providing empirical data on patient perspectives (Table 1). We limited inclusion to studies published after 2009 to align with the major advances in AI and telemedicine over the past 15 years, during which AI became increasingly integrated into both health care and broader societal applications, and telemedicine saw widespread adoption across global health systems [5,6]. This timeframe was chosen to capture evolving patient expectations and technological standards that more accurately reflect current experiences. Ten additional papers identified through reference list screening of included studies were incorporated to provide background context, bringing the total to 48 papers. Our search revealed no publications directly comparing patient perspectives of AI-integrated telemedicine in dermatology, highlighting a gap in current literature.

**Table 1.** Inclusion study characteristics.

Reference	Study aim	Setting	Sample, n	Study type
Abeck et al [12]	To investigate the impact of teledermatology on patient care by characterizing consultations on a direct-to-consumer telemedicine store-and-forward platform.	Retrospective data obtained from Wellster Healthtech Group in Germany.	n=1999 (retrospective analysis); n=166 (8.3%) (follow-up survey)	Retrospective Analysis and Survey
Asabor et al [13]	To examine the experience of patients and physicians with teledermatology during the COVID-19 pandemic.	Patients seen via Epic MyChart synchronous video visits.	n=548	Survey
Balakrishnan et al [14]	To explore patient satisfaction with teledermatology and 2 distinct teledermatology models.	Patients seen at the Atlanta Veterans Affairs Medical Center.	n=100	Survey
Choi et al [15]	To understand patient perceptions toward teledermatology.	Patients or their caregivers at an academic tertiary dermatologic center in Singapore.	n=913 (survey); n=26 (2.9%; in-depth interviews)	Survey and Semistructured Interview
Chow et al [16]	To explore patients’ perceptions of a teledermatology service linking public primary care clinics to a national specialist dermatology clinic.	Five separate dermatology clinics in Singapore.	n=21; patients aged 22‐72 years; 14 (65%) male; diagnoses: 11 (52%) rashes, 4 (19%) pigmented lesions, 3 (14%) itching, and 2 (10%) dry skin	Qualitative Interview
DeVries et al [17]	To assess perceptions and experiences with teledermatology visits in the context of the COVID-19 pandemic.	Patients of a South Dakota dermatology practice.	Not specified	Survey
Ford et al [18]	To evaluate the impact of a web-based Collaborative Connected Health model compared to in-person care on access to specialty care for psoriasis management.	Patients from outpatient clinics and general adult populations in California and Colorado.	n=300; 151 (50.3%) male; mean age: 49 years; 190 (63.2%) White; 101 Hispanic or Latino (33.8%); 13 (4.4%) uninsured	Randomized Controlled Trial
Frühauf et al [19]	To explore patient satisfaction with video consultations for inflammatory skin conditions in a dermatology outpatient setting.	A teaching hospital in Wales, United Kingdom, has an outpatient dermatology clinic.	n=48; 35 (72%) female; age range 13‐80 years	Survey
Ghani et al [20]	To identify demographic and behavioral factors associated with patient interest in using teledermatology.	Data from the Health Information National Trends Survey 4, cycle 4 of the National Cancer Institute.	n=3677; 1338 (36.4%) male; age 50‐64 (31.8%); 1419 (38.6%) college or higher education; 1894 (51.5%) non-Hispanic White; 963 (26.2%) income>US $75,000	Survey
Gnanappiragasam et al [3]	To assess patient satisfaction and preferences between face-to-face and remote (telephone or video) consultations in dermatology settings.	Two dermatology centers in the United Kingdom.	n=156; 78 (50%) female; mean age: 53.3 years; divided into new and follow-up groups	Survey
Goessinger et al [21]	To investigate the perspectives of patients and dermatologists after skin cancer screening by human, artificial, and augmented intelligence.	The University Hospital Basel, Switzerland.	n=205; mean age 54.8, SD 13.6 years; 109 (53%) male	Survey
Hadjieconomou et al [2]	To explore patient satisfaction with video consultation within a dermatology outpatient clinic setting for preselected inflammatory skin disorders.	Dermatology outpatient clinic in Wales, United Kingdom.	n=48; 35 (72%) female; age range 13‐80+ years; 4 (8.5%) aged >65 years	Survey
Handa et al [22]	To analyze patient and physician experiences and acceptability of teledermatology over a 6-month period.	A tertiary care center in North India.	n=5229; mean age 33.60, SD 16.99 years; 2714 (51.9%) male	Survey
Horsham et al [23]	To investigate the factors that determine consumers’ comfort and willingness to share 3D total-body images for research, AI^a^ development, clinical, and teaching scenarios.	Online video-based consumer forum for consumers of 3D total-body imaging studies at the UQ^b^ Dermatology Research Center.	n=39	Survey
Hsueh et al [24]	To assess patient satisfaction with a store-and-forward teledermatology.	27 Veterans Integrated Service Network; 20 clinics in Alaska, Idaho, Oregon, and Washington.	Face-to-face: n=196; 190 (97%) male; mean age 71 years; Teledermatology care: n=504; 464 (92%) male, mean age 65 years	Survey
Hwang et al [4]	To review patient satisfaction with the use of teledermatology since the COVID-19 pandemic.	Not applicable	32 studies: 13 randomized controlled trials, 14 narrative reviews, 5 systematic reviews	Narrative Review
Jutzi et al [25]	To investigate the hopes and fears of patients with and without a history of melanoma toward the use of AI in skin lesion diagnostics.	Web-based questionnaire using LimeSurvey sent to university hospitals in Germany.	n=298; 225 (75.5%) female; 123 (41.3%) aged 46‐60 years; 121 (40.6%) with a university degree	Survey
Kawsar et al [26]	To explore patients’ perspectives on the use of AI as part of their skin cancer management pathway.	A teledermatology skin cancer clinic at Chelsea and Westminster Hospital, London, United Kingdom.	n=268; 154 (57.5%) female; aged 18‐93 years. Skin type: 218 (81.3%) Fitzpatrick type I-II	Randomized Controlled Trial and Survey
Kohn et al [27]	To evaluate the acceptance of synchronous telehealth for pediatric dermatology.	Children’s Hospital Colorado Pediatric Dermatology.	n=125; mean age 9.2 years; 57 (45.5%) male; 48 (38.5%) new patient	Survey
Lim et al [28]	To obtain opinions of patients on the use of AI in a dermatology setting, when aiding the diagnosis of skin cancers.	Dermatology outpatient skin cancer clinics in 2 United Kingdom hospitals.	n=603; 314 (52%) female; age range: 18‐100 years; 452 (75%) new referrals; 555 (92%) concerned about skin cancer	Survey
Lowe et al [29]	To evaluate the clinician and patient/parental perspective of a pediatric dermatology clinic via voice calls and emailed images in comparison to traditional face-to-face clinics.	United Kingdom single-center cohort of pediatric dermatology patients managed during the COVID-19 pandemic.	n=116; mean age 8.47 years; 28 (24%) new patients; 87 (75%) cases of inflammatory dermatoses	Survey
Ly et al [30]	To understand individuals’ perceptions of sharing their images for AI.	Adult United States respondents via Amazon Mechanical Turk.	n=1010; mean age 36.5 years; 566 (56%) male; 717 (71%) White; 851 (84.3%) employed	Survey
Maul et al [31]	To investigate the acceptance of and satisfaction with telemedicine.	One secondary and 2 tertiary referral centers for dermatology in Switzerland.	n=512; 273 (53.3%) male, mean age 49.5 years	Survey
Moore et al [32]	To evaluate patient satisfaction with university medical center’s video-based teledermatology service.	Penn State’s Dermatology Department	n=171; 118 (69%) female, 154 (90%) non-Hispanic	Survey
Munoz et al [33]	To investigate and compare patient satisfaction with recorded video counseling vs traditional, in-office counseling.	Not specified	n=16; video counseling: n=11 (68.8%); face-to-face counseling: n=5 (31.3%)	Survey
Naik [34]	To gain a global perspective on the experiences of patients and health care staff who adapted to teledermatology during the COVID-19 era.	Recruitment through social media and WhatsApp groups.	n=653	Survey
Nelson et al [1]	To explore how patients conceptualize AI and perceive the use of AI for skin cancer screening.	Brigham and Women’s Hospital and the melanoma clinics at the Dana-Farber Cancer Institute.	n=39; mean age: 53.3 years; 21 (54%) female; 37 (94%) non-Hispanic White; 16 (42%) graduate or professional degree	Qualitative Interview
Qun Oh et al [35]	To examine patients’ perceptions of teledermatology and identify barriers to its adoption.	Outpatient dermatology clinic at a tertiary academic medical hospital in Singapore.	n=997; 508 (51%) female; 489 (49%) aged ≥60 years)	Survey
Pathoulas et al [36]	To compare patient satisfaction between telemedicine visits and in-office visits in a specialty hair loss clinic.	Patients who received either an in-office or telemedicine hair loss new patient consultation by a single provider.	n=40; 29 in-office (72.5%), 11 (27.5%) telemedicine	Survey
Ramjee et al [11]	To assess patient satisfaction with telephone consultations compared to face-to-face consultations in secondary-care dermatology during the COVID-19 era.	A single dermatology center in London, United Kingdom.	n=74; 43 (58.1%) female; median age of 52 years	Survey
Richey et al [37]	To evaluate patients’ perspectives and preferences regarding teledermatology for cosmetic acne scar treatment.	Patients at FORMEL Skin in Berlin, Germany.	n=842	Survey
Ruggiero et al [38]	To assess how patients with acne subjectively experienced teledermatology visits.	The Acne Care Center, Dermatology Unit, University of Naples Federico II, Italy.	n=52; 28 (53.9%) female; mean age: 22.5 years.	Survey
Sangers et al [39]	To explore the perceived barriers and facilitators to using mHealth^c^ AI apps for skin cancer screening.	The Netherlands	n=27; median age 25 years; 18 (68%) female; 11 (41%) had previous experience with mHealth apps; 4 (15%) had a history of skin cancer	Survey
Stratton et al [40]	To assess patient preferences regarding the use of postprocedural photographs compared with in-person follow-up.	The University of Alabama at Birmingham Department of Dermatology.	n=150; 89 (59.5%) male	Survey
van Erkel et al [41]	To evaluate the perceived quality of follow-up telephone consultations of multiple medical disciplines during the COVID-19 pandemic.	Large university hospital in the Netherlands.	n=82; 44 (54%) female, mean age: 59.1 years	Semi-structured Interview
Wortman et al [42]	To evaluate the pandemic’s implications on patients with psoriasis, focusing on access to information, consultation methods, patient satisfaction, disease control assessment, and treatment management.	Multicenter survey from 4 Dutch hospitals during the second wave of the pandemic.	n=551; 309 (56%) male, median age: 59 years old, median disease duration: 25 years old	Survey
Wu et al [43]	To gather opinions from a diverse dermatology patient population on AI use in dermatology and establish a specific accuracy at which patients would be comfortable receiving a diagnosis solely from an AI tool.	Adult patients who visited the University of Texas Southwestern Medical Center Dermatology.	n=141; 73 (52%) male; mean age: 55.3; 79 (56%) non-Hispanic white; 55 (39%) household income US $50,000–US $99,999	Survey
Yadav et al [44]	To assess patient perception and satisfaction with a smartphone-based teledermatology service initiated during the COVID-19 pandemic.	The Department of Dermatology and Venereology, All India Institute of Medical Sciences (AIIMS), New Delhi.	n=201; 109 (54.2%) male, mean age 38.4 (SD 15.7) years	Survey

a AI: artificial intelligence.

b UQ: University of Queensland.

c mHealth: mobile health.

Three reviewers independently extracted predefined attributes from each paper. Disagreements were resolved through discussion until a consensus was reached. Literature primarily focusing on technical aspects of AI or telemedicine implementation, without substantial discussion of patient perspectives, was deemed outside the scope of this review.

While our review applied structured screening and thematic synthesis similar to a scoping review, we selected a narrative review approach to enable conceptual interpretation of patient perspectives across diverse study types. This approach allowed us to synthesize findings not only by outcome themes but also by behavioral drivers and contextual patterns. The narrative format also guided our inclusion criteria, enabling us to incorporate both quantitative and qualitative studies that offered insight into patient perceptions, even when methods or outcome measures were heterogeneous. This review was not preregistered, and no formal checklist such as PRISMA (Preferred Reporting Items for Systematic Reviews and Meta-Analyses) was used, although we followed structured screening procedures to enhance methodological transparency.

### Ethical Considerations

This paper is a narrative review and does not involve primary data collection with human or animal participants. Therefore, institutional review board approval and informed consent were not required. The study adhered to the principles of the Declaration of Helsinki and followed JMIR Publications’ ethical guidelines for secondary research and literature reviews.

## Results

### Overview

**Figure 1.**
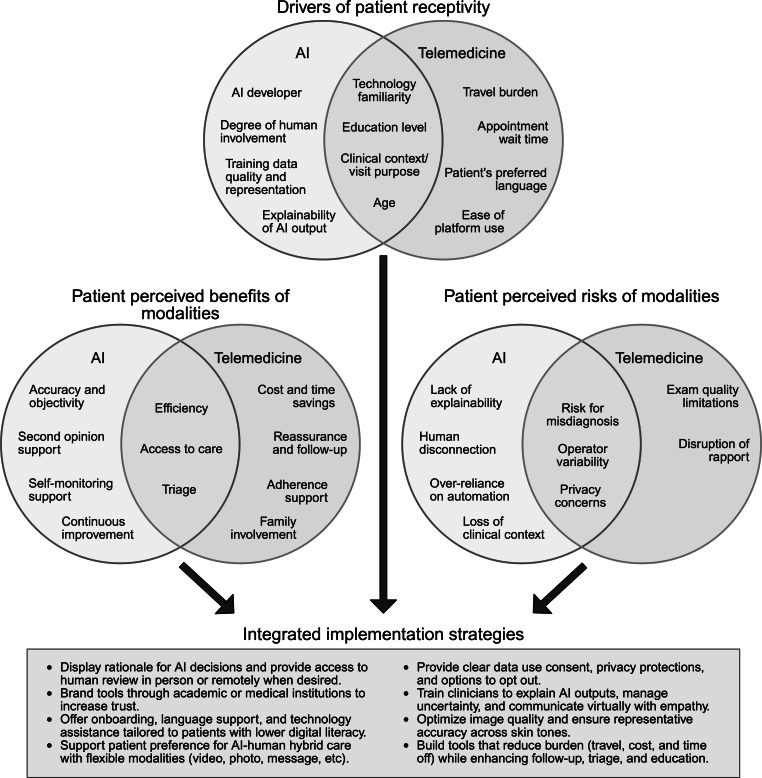
A patient-informed framework for artificial intelligence and telemedicine integration in dermatology. AI: artificial intelligence.

The literature revealed several distinct themes regarding patient perspectives on AI and telemedicine in dermatology, also known as “teledermatology.” The following sections examine these themes for each technology, including patient willingness to use, perceived benefits and risks, barriers to implementation, and conditions necessary for successful integration into dermatology. Each section compares perspectives between AI and teledermatology while highlighting how these viewpoints might inform future implementation strategies. Tables2  3, located after the Results section, summarize key themes from patient perspectives on AI and teledermatology, respectively. The column categories of these tables were adapted from Kalkman et al [45]. Figure 1 presents a conceptual framework that synthesizes these findings to guide integrated implementation approaches.

**Table 2.** Patient perspectives on artificial intelligence in dermatology.

Perceived benefits	Perceived risks	Barriers to using	Factors affecting willingness to use	Conditions for acceptance
Increased diagnostic speed [1] and accuracy [21] due to the ability of AI to learn, evolve, and draw on larger data and experience than humans [1,28]	Lack of verbal and nonverbal communication [1]	Inaccurate or limited training sets [1]	Familiarity with AI is significantly associated with a positive view (OR: 17.8; *P*<.01) [43]	AI must be (mean 12.9%, SD 8.1%) more accurate than dermatologists [43]
Enhanced health care access [1]	Increased patient anxiety [1]	Age-related differences in familiarity [43]	Age 40‐59 years associated with decreased AI familiarity (OR: 0.21, *P*<.01) [43]	Integration with human oversight [1,28]
Potential for earlier detection of skin cancer and lifesaving outcomes [1]	Loss of human interaction and human emotion [1,25]	Limited explanation of AI decisions [1]	Higher education levels are associated with increased willingness to share images with AI [30]	Clear privacy policies and safeguards [23]
Promotes patient engagement in self-examination [1]	Privacy concerns [1,39]	Concern about AI’s inability to provide emotional support [1]	97% (289/298) of respondents with a previous history of melanoma support AI use in medicine compared to 91% (271/298) of patients without melanoma (*P*=.03) [25]	System validation by medical professionals [1]
Reduced health care costs [1]	Patient loss to follow-up [1]	Anxiety about receiving a diagnosis without human support [1]	Comfort with technology and social media sharing [30]	Clear guidelines for image control and use in AI [23]
More convenient, consistent, and objective diagnosis [1]	Nefarious use of AI [1,25]	Potential for false positives and false negatives [1]	Trust in a developing institution affects willingness [23].	Need for demonstrated effectiveness and openness of AI use [28]
Unburdening of the health care system [1]	Human deskilling [1,25]	Lack of in-person physical examination [1]	No association between age and acceptance of an AI-only diagnosis, age and preferred AI involvement by diagnosis severity, or prior skin cancer diagnosis and reluctance to use AI for diagnosis [28].	Assurance that AI will not replace discussion with a human dermatologist [28]
Physicians can learn from AI-based^a^ systems and direct comparison may motivate specialists to continue to improve performance [25]	Potential misdiagnosis or inaccuracy [28,39]	Operator dependence [1]	Patients felt a greater sense of safety with AI when it worked in tandem with a dermatologist rather than independently [21].	Endorsement from health care providers and government regulating bodies [39]
Improved triage efficiency [1]	Inability to answer follow-up questions [1,25]	Inability to assess treatment options [1]	Patients did not believe AI could answer follow-up questions, discuss treatment options, educate, or reassure [1]	Usable by all ages [39]
Reduced patient anxiety [1]	Lack of context in AI decisions [1]	Inability to educate or reassure patients [1]	More trust if AI applications are set up by dermatologists rather than companies [1].	Low cost of use [39]
Acts as a second opinion to refer to a dermatologist [1,28]	Concerns about image control and secondary use [23]	Lower agreement scores for AI guiding general practitioners in Fitzpatrick IV-VI (44.6/100) vs Fitzpatrick I-II (74.8‐81.4/100) [26]	—^b^	—
Can perform skin cancer screening from home, monitor skin lesions over time, and integrate with existing skin cancer care [39]	Data privacy risks for sensitive biometric data [23]	Limited knowledge about the use and functionality of AI [39]	—	—
More reliable and less subjective diagnoses [25]	—	Lack of integration into the health system and therefore perceived lack of value [39]	—	—
—	—	Lack of reliability of AI app developers for skin cancer screening [39]	—	—

a AI: artificial intelligence.

b not applicable.

**Table 3.** Patient perspectives on telemedicine in dermatology.

Perceived benefits	Perceived risks	Barriers to using	Factors affecting willingness to use	Conditions for acceptance
Reduced wait times and increased efficiency [3,4,11,13,15,16,18,24], reduced travel needs [2,3,11-13,18,27,29 ], reduced work and school absence [2,3,13,18,29], and savings on parking costs [3,18]	Concerns over the quality of clinical examination [3,15,29,34,44]	Technology limitations, poor internet connectivity, and digital health literacy challenges [3,32,34,35]	Distance from clinic (preference increased with greater distance*; P*=.04) [40]	Careful selection of patients who are better able to navigate technology for telemedicine appointments [3,4]
More efficient triage for acne and skin cancer [4]	A doctor may miss important details [11,40,44]	Less social and natural interaction, reduced ability for the clinician to feel skin pathologies [11]	Rural patients rated their experiences higher than urban counterparts, suggesting satisfaction reflects regional availability of care [5]	Clear preappointment instructions [4]
Improved medication compliance and treatment adherence [4]	Technical difficulties affecting care [3,16,24,34]	Need for assistance taking photos [40]	Previous telemedicine experience increased acceptance [5]	Adequate follow-up care systems [24]
No need to find childcare [27,34]	Privacy concerns [16,34,40]	Sensitivity of health condition [3]	Trusting web-based health information and previous experience sharing medical data on an app or on the web [18,20]	Digital divide factors addressed to ensure equitable access across diverse populations [20]
Improved access to care [2,24]	Decreased quality of care [11,35]	Variable digital literacy levels [29]	Patients preferred chronic conditions over initial consultations [18]	Educational materials provided [24]
Better family involvement [2]	May still require an in-person visit [40]	Cost issues for some patients [34]	About one-third of patients with alopecia maintained a strong preference for in-person evaluation [36]	High-quality image requirements and technical quality of telemedicine video [3,4]
Ability to send concerns anytime [40]	Inadequate follow-up care [24]	Difficulty with photos of hard-to-reach areas for photo-based teledermatology clinical evaluation [18]	Patients with acne preferred teledermatology, whereas patients with possible malignant lesions preferred in-person visits [17]	Trust-building measures [20]
Quicker reassurance and follow-up [40]	Inability to perform labs and procedures [27]	The platform is not user-friendly enough for a mobile interface [18]	Older age increased the likelihood of agreeing to use telemedicine again in some studies [14], but decreased it in others [17]	—^a^
Reduced patient anxiety [2]	Lack of personal element [3,29,44]	Language barriers [3,18]	No significant differences in patient satisfaction and comfort with telemedicine use based on age or visit (*P*=.79*; P*=.90) [36]	—
70.3% (105/150) believed it would improve care; 27.6% (41/150) believed no change in care [40]	Substandard physical examinations [3,4,11,27,29,34]	Privacy concerns specifically about sending personal information [40]	Telemedicine became preferable with an in-person wait time of 6.89 months [36]	—
Useful for monitoring systemic therapies [4]	Limited visual cues and body language [11,29]	Technical difficulties were a common reason for preferring face-to-face consultations [3]	53% (44/83) would consider remote over face-to-face if appointment expedited [3]	—
Valuable for various conditions: acne, atopic dermatitis, and psoriasis [4,38]	Reduced quality of patient-clinician communication [29] and a lower chance of discussing other skin concerns [3]	Patients who had to travel longer distances were not significantly more likely to think that teledermatology is more convenient than face-to-face appointments [14]	The quality of video-based exams, images [4], and audio [2]	—
Video allows family to view consultation multiple times and on their own time [33]	Concerns about improper treatment recommendations due to lower accuracy during telemedicine interaction [24]	—	Strong emotional support and rapport with physicians [18]	—
Less exposure to infection risk [2,34]	Inability to relay parental anxiety about pediatric care as effectively via telemedicine vs face-to-face [29]	—	Older patients were more likely to always prefer an in-person wound check compared to younger patients (*P*<.01) [40]	—
Increased time to spend with family [34]	Technology literacy limitations [3,34]	—	Age was not a factor in willingness to use [22]	—
—	2.1% (n=3/150) of participants perceived a negative impact on care [40]	—	Experiencing technical difficulties during a previous telemedicine encounter [17]	—
—	—	—	Previous exposure to video conferencing and higher education levels [35]	—
—	—	—	Some patients preferred phone consultations for discussing sensitive topics to avoid eye contact [41]	—
—	—	—	47% (443/942) more willing to use teledermatology during the pandemic vs 26% (245/942) before the pandemic [15]	—

a Not available.

### Willingness to Use AI Versus Telemedicine in Dermatology

The literature shows that patients exhibit hesitancy toward AI-based diagnoses without dermatologist interventions. In a survey conducted by Wu et al [43], if a dermatologist and an AI model made different diagnoses, the majority of patients (119/141, 84.4%) favored the dermatologist’s diagnosis. They also found that about 14.9% (21/141) of patients expressed “complete unwillingness” to be evaluated by AI alone [43]. In contrast, Lim et al [28] found that patients would be happy for their general practitioner to use AI to make dermatologist referral decisions (235/603, 39%).

In suspected skin cancer cases, patient trust in human expertise remained high, with a significant majority (524/603, 86.9%) of patients strongly preferring a dermatologist’s diagnosis, and only 12.1% (73/603) willing to accept a diagnosis made solely by AI [28]. Despite hesitancy toward standalone AI diagnoses, the Wu et al [43] survey data revealed that most patients (96/141, 68.1%) preferred dermatologists to use an AI model that could provide differential diagnoses based on a photograph at the point of care rather than working alone. This pattern suggests patients prefer AI as a decision-support tool that enhances rather than replaces clinical judgment.

Patients’ acceptance of dermatology-based AI tools showed overall mild concerns for data sharing privacy. While Lim et al [28] found that 87% (508/584) of patients were willing to share their patient images for AI training and to help other patients, Ly et al [30] determined that patient comfort levels declined when more facial or sensitive areas were in question. For example, 81% (820/1010) of patients were comfortable with sharing images of their hands, 70% (710/1010) with images of their face, 58% (326/563) with images of male genitals, and 47% (209/447) with female genitals [30].

The willingness to share sensitive or identifiable images was also dependent on the AI tool’s development, and Horsham et al’s study [23] concluded that patients were more willing to share pictures with university-developed tools than those that were industry-developed. The preference for university-developed AI tools over commercially-developed ones likely reflects broader perceptions of institutional trust. Patients may associate academic institutions with stricter data privacy protections, ethical oversight, and transparency in tool development, whereas commercial tools may raise concerns about profit motives and potential misuse of sensitive health information. These perceptions reiterate the importance of transparent development processes, ethical governance, and academic partnerships in cultivating public trust in AI technologies.

Despite the rising popularity of teledermatology appointments, patient preferences for consultation methods remain mixed (Table 3). Balakrishnan et al [14] identified that older age was associated with an increased likelihood of using telemedicine for follow-up appointments, whereas studies by DeVries et al [17] and Choi et al [15] found a negative association between age and willingness to use teledermatology services for follow-up appointments.

In studies with patients who experienced both face-to-face appointments and teledermatology, preferences widely varied. In Gnanappiragasam et al’s study [3] of patients with a mean age of 53.3 years, 61% (97/156) preferred face-to-face for future consultations, while 39% (60/156) did not have a preference for appointment modality. Meanwhile, Hsueh et al [24] reported that 66% (332/503) of veterans, with a 92% (464/503) male population and a mean age of 71 (SD 17) years, preferred teledermatology over face-to-face. Finally, Hadjieconomou [2] had a 72% (34/48) female demographic, with 91.5% (44/48) younger than 65 years and an 8% (4/48) preference for face-to-face visits.

These contrasting findings related to participant age likely reflect context-specific factors. For instance, the high telemedicine acceptance among older male veterans in Hsueh et al’s study [24] may be shaped by functional limitations (such as mobility impairments), structured support from the Department of Veterans Affairs system, or previous exposure to digital tools in service settings. In contrast, Hadjieconomou’s [2] younger, predominantly female population may have expressed reluctance due to concerns about privacy, self-image on video, or decreased rapport during teledermatology consultations. These findings suggest that age, gender, previous technology exposure, and health status may influence teledermatology acceptance through competing functional and technological factors.

Regarding positive feelings toward telemedicine, the prospective study conducted by Lowe et al [29] reported that patients (98%, 41/42) with telemedicine consultations felt their concerns were addressed during consultations. Ford et al’s [18] randomized controlled trial demonstrated that 70% (210/300) believed telemedicine improved care, 27% (82/300) reported no change, and 2% (6/300) perceived a negative impact.

Adding to these findings about patient preferences, Frühauf et al [19] demonstrated strong patient acceptance of teledermatology among patients with psoriasis, with 90% (n=9/10) reporting they felt “in good hands” with remote care while experiencing a more flexible lifestyle. The same study found that 80% (n=8/10) of patients considered teledermatology a viable alternative to in-person consultations, suggesting high levels of patient confidence in remote care delivery [19]. Finally, a small subset of patients in a Dutch study who used chat or email consultations graded their experience as a 9 out of 10 in satisfaction [42].

However, underlying these positive ratings, Table 3 shows that a few studies reported that patients expressed concerns about the lack of patient-physician connection during initial consultations [3,29,44]. This concern appears to be related to patients’ previous experience with technology, as Qun Oh et al [35] found that patients were more likely to decline telemedicine if they had minimal exposure to video conferencing. This technology-related hesitation highlights the importance of gradually exposing patients to telemedicine platforms to build familiarity and comfort with remote care delivery.

A final interesting observation was that patient preferences for telemedicine varied significantly based on their specific dermatological conditions and needs. Handa et al [22] reported that the highest levels of satisfaction (3419/5229, 65.4%) with telemedicine were seen in patients with infectious dermatologic manifestations. However, for chronic disease management, Ford et al [18] found that 65% (195/300) of patients surveyed preferred in-person follow-ups.

Furthermore, findings from a survey by DeVries et al [17] demonstrate that patients with acne had a strong preference for teledermatologic visits, whereas those with possible malignant lesions strongly preferred an in-person visit. Another study comparing video counseling to in-office counseling for acne isotretinoin initiation found no significant difference in patient satisfaction scores across multiple domains, including comfort starting isotretinoin and concerns about side effects [33]. Ruggiero et al [38] reported specific aspects of teledermatology that patients with acne were highly satisfied with, including dermatologist attention (48/52, 92%), quality of time spent with the dermatologist (45/52, 87%), and the treatment received (37/52, 71%). These variations in willingness to use telemedicine reflect patients’ risk assessment preferences, with higher-stakes conditions driving demand for direct physician contact.

Collectively, patient preferences for both AI and teledermatology are influenced by factors such as demographic characteristics, previous technology experience, the level of clinician involvement, and specific dermatological needs. Understanding these preference patterns helps design patient-centered implementation strategies that maximize patient acceptance and engagement.

### Perceived Benefits of AI Versus Telemedicine in Dermatology

Many facets of patient care could be impacted by the use of AI in dermatology, and patients’ motivations for its implementation are optimistic. As shown in Table 2, a qualitative study by Nelson et al [1] found that some of the primary patient values of AI relate to its potential for quicker diagnoses (29/48, 60%), greater ease of health care access (29/48, 60%), and increased triage efficiency (14/48, 29%) [1]. Approximately 35% (17/48) also associated AI with reduced health care costs. However, this survey focused on using AI as a skin cancer screening tool, and a majority (32/48, 67%) of participants had a history of melanoma or other skin cancer. Given this context, future research stratifying patient responses by disease history would be valuable.

Additional research by Sangers et al [39] provided valuable insights into the practical benefits that patients associate with using AI, specifically for skin cancer screening. These included the ability to perform skin cancer screenings from home and monitor lesions over time, giving patients a better sense of involvement in their dermatological health care. As provided in Table 3, patients surveyed by Goessinger et al [21] following AI-assisted skin cancer screening reinforced these positive perspectives, believing that AI enhances diagnostic performance (195/205, 95.5%). These patient perceptions of the potential benefits of AI reflect some of AI’s greatest strengths as it integrates into dermatological health care.

Meanwhile, telemedicine addresses several logistical challenges patients face when accessing dermatological care. Efficiency was a primary benefit noted by patients, and Abeck et al [12] reported that the most frequent reason for using teledermatology was shorter waiting times for appointments (103/166, 62%). This finding was reinforced by Pathoulas et al [36], who found that patients preferred a telemedicine visit with a 2‐to 3-week wait time over an in-office visit with a wait longer than an average of 6.89 months.

Table 3 highlights another significant advantage of telemedicine cited by patients across studies—its reduction in travel and parking time and costs [2,3,11-13,18,27,29], which is particularly important for patients living in remote areas to improve access to care (Table 3). Interestingly, 3 of these studies evaluated patient perceptions during the COVID-19 pandemic [11,13,29]. It is possible that many positive opinions were driven by limited in-person care options, further supported by Choi et al [15] finding that telemedicine support increased during the pandemic and then decreased after movement restrictions eased. The advantage of reduced travel time is complemented by telemedicine’s potential to minimize absences from work or school [2]. Patients also appreciate teledermatology’s flexibility and the ability to send dermatological concerns at any time [40]. Uniquely, Hadjieconomou [2] found that 71% (34/48) of patients valued its reduction in the risk of infection exposure, and 55% (26/48) appreciated more feasible family involvement during the telemedicine consultation.

Improved treatment outcomes also emerged as a noteworthy patient-reported benefit of teledermatology. A randomized controlled trial by Ford et al [18] found that telemedicine facilitated better psoriasis management, as patients could submit photos and receive real-time updates to their treatment plans based on disease progression. Similarly, participants from a Swiss questionnaire expressed their positive perceptions toward telemedicine for minor skin problems [31]. A German survey also found that patients believed telemedicine represented a useful and underused screening tool for cosmetic dermatology before physician evaluation [37]. These results demonstrate that, from the patient perspective, teledermatology serves as an effective initial management and screening tool for chronic, minor, and cosmetic dermatological concerns, which may evolve to additional disease contexts as patients build trust with the platform.

Overall, patients report similar perceived benefits in the usage of AI and telemedicine services in dermatologic practice, ranging from improved accessibility to enhanced care outcomes.

### Perceived Risks of AI Versus Telemedicine in Dermatology

Patients perceived the greatest risks of integrating AI into dermatologic practice to be its limited communication abilities and inherent constraints as an algorithmic tool. Table 2 highlights that patients’ primary concerns about AI center on its limited communication abilities, with 40% (19/48) noting the absence of nonverbal communication as key risks [1]. Loss of social interaction (18/48, 38%) was similarly identified as a risk, as patients doubted AI’s ability to respond appropriately to emotional distress [1]. In terms of verbal communication, patients emphasized AI’s limited capacity to provide education or answer follow-up questions [1]. As AI advances in its real-time interaction capabilities, this particular concern may gradually diminish.

Table 2 highlights patients' expressed concerns about potential misdiagnoses by AI, including false negatives and positives, limited training datasets, lack of physical examination, and operator error [1]. As mentioned in the “Willingness to Use” section, patients exhibited hesitancy toward AI’s use as an independent diagnostic tool [28]. These concerns for AI as a diagnostic tool greatly contrast with patient perceived risks of AI as a screening tool, which primarily focused on AI’s lack of empathy [1]. As AI begins playing a larger role in dermatologic care, clearly communicating the intended purpose of the technology—whether for screening or diagnosis—may help alleviate patient hesitancy.

Data security emerged as another potential risk, with patients in the Nelson et al study [1] highlighting concerns about loss of privacy (14/603, 29%) and nefarious use of AI (11/603, 23%). These hesitations are valid and highlight the need for dermatologists to adopt transparent AI tools and proactively communicate their limitations, privacy safeguards, and intended roles in care to build trust and mitigate patient concerns.

In response to teledermatology, patients perceived substandard physical examinations as the greatest risk, and numerous studies reported patient concerns regarding the quality of teledermatology complete skin examinations, especially for those being monitored for skin cancer [3,4,11,27,29,34,41] (Table 3). Respondents to an Alabama survey of 150 patients expressed concerns that doctors might miss critical details in a teledermatology setting (150/235; 63.8%). They also noted that an in-person check might still be needed after teledermatology care (32/235, 13.5%) [40].

Hsueh et al [24] reported similar apprehensions, as shown in Table 3, finding that patients were concerned about improper treatment recommendations due to lower diagnostic accuracy during telemedicine interactions. Qualitative interviews by Chow et al [16] also revealed that camera quality was a key reason why patients were concerned with diagnostic accuracy. Given that some studies describe patient beliefs that AI can improve diagnostic accuracy, reliability, and efficiency [1,25,28], integrating AI with teledermatology could help mitigate concerns about the limitations of remote physical examinations. However, some telemedicine limitations remain beyond AI’s scope, as surveys for parents of patients with pediatric conditions identified that several required laboratory tests and procedures could only be performed in person [27].

Similar to AI-related risks, limited personal elements also contribute to patients’ concerns about reduced quality of care via virtual telemedicine platforms [3,29] (Table 3). Specifically, patients in a UK survey reported that teledermatology consultations lacked nonverbal cues, which led to worse patient-physician understanding and weakened rapport [11]. Similar sentiments were expressed through qualitative feedback on telemedicine experiences in a study by Yadav et al [44], where patients noted concern for the lack of personal touch during the consultation. In a pediatric cohort, parents of patients reported that telemedicine was less effective in easing their anxiety compared to face-to-face visits, and 52% (60/116) of surveyed participants expressed significant dissatisfaction with the telephone clinic [29].

Overall, patients share similar concerns about both AI and teledermatology centered around diagnostic accuracy limitations and reduced human connections (Figure 1). Thus, integration of these technologies must focus on preserving existing human interaction. Teledermatology faces additional scrutiny of physical examination quality, while AI elicits concerns about emotional responsiveness and data security.

### Barriers to Adoption of AI Versus Telemedicine in Dermatology

Beyond perceived risks, patients identify several practical and trust-related barriers that may limit the successful integration of AI and teledermatology into dermatologic care. Table 2 shows that patients are concerned with poor training datasets for AI and the necessity for clinicians to still interpret AI results to develop effective treatment plans [25]. Patients in qualitative interviews by Sangers et al [39] specifically mentioned that limited knowledge about the use and functionality of AI was a barrier to its integration.

Patient trust in dermatologic AI services may also be undermined by data security risks. As discussed in “Willingness to Use,” patients would much rather share sensitive or identifiable images with university-developed AI versus private industry-developed systems [23]. Notably, only 15% (6/39) of these respondents answered that they had a “high” level of knowledge about AI, as opposed to 72% (28/39) who selected “low” or “moderate.” Therefore, patient perceptions of AI security and privacy could significantly change with increased familiarity with AI technology and its privacy protections.

Finally, a survey on patient acceptance of AI in skin cancer diagnostic pathways revealed that Fitzpatrick skin type strongly influenced patient agreement scores regarding the use of AI to assist their general practitioner (*P*=.02) [26]. Patients with darker Fitzpatrick skin types IV–VI reported a median agreement score of 44.60 out of 100, significantly lower than those with Fitzpatrick types I (79.89/100), II (81.39/100), and III (74.77/100) [26]. Therefore, AI training datasets must be representative of all skin types to ensure that AI operates equitably and fosters trust among patients.

Regarding teledermatology, patients mentioned technological limitations as the primary barrier to adoption [3,20,32,34] (Table 3). This barrier contrasts with the main perceived risk relating to substandard physical examinations, suggesting that it may be more difficult to circumvent technological limitations. One survey revealed that patients with lower satisfaction scores were significantly more likely to have experienced technical difficulties or to perceive their teledermatology-based physical examination as unsatisfactory [32]. The literature shows that, for patients with limited digital literacy, nonuser friendly or uninviting teledermatology platforms may exacerbate challenges with teledermatology visits [18,29]. Therefore, clinicians using teledermatology must ensure that digital services are simple to navigate and offer extensive troubleshooting for technology-related problems.

Other logistical barriers emerged for teledermatology use and adoption. Table 3 includes 2 studies noting that patients often struggled to take photos of hard-to-reach areas for teledermatology visit evaluation, occasionally requiring assistance from others [18,40]. This finding expands on previous data that describes patients’ hesitancy to share images of certain body parts [30], further indicating that image location plays an important role in patient comfort. In addition, a retrospective survey on telephone consultations found that teledermatology visits were less preferred by patients due to reduced natural social interactions and the clinicians’ inability to physically examine the patients’ skin [11]. Table 3 also highlights patients’ emphasis on the importance of visually seeing the clinician as a care preference [11].

In summary, barriers to both AI and teledermatology adoption stem from certain technological limitations and trust concerns and are important to address before technological convergence or implementation.

### Conditions for Using AI Versus Telemedicine in Dermatology

For AI and teledermatology to be successfully integrated into dermatological care, patients have distinct considerations and requirements. Patients emphasize the need for dermatologist oversight for AI system model validation and to ensure that AI would not replace human discussion [1,25,28,43]. Current AI models that use patient images have brought up concerns about the adequacy of existing guidelines and policies around AI [23]. Consequently, patients desire safeguards and transparency of the tools to guarantee clear AI privacy policies, secondary uses of data, and AI’s effectiveness [23,28,46]. As shown in Table 2, patient acceptance of AI is heavily contingent on demonstrated superiority, with patients requiring AI to be a mean of 12.9% (SD 8.1%) more accurate than dermatologists before accepting standalone AI evaluations [43]. Most importantly, patients indicated that endorsement from their dermatologist and government regulatory bodies would promote their acceptance of AI use in dermatological care [39].

To improve the adoption of teledermatology services, patients outlined several practical and operational factors surrounding their appointments. For example, patients expressed that preappointment teledermatology educational materials and adequate follow-up care systems are important considerations for use [4,24]. Technical difficulties were frequently cited as a reason for preferring face-to-face consultations [3]. For this reason, patients noted that high-quality images and video should be required for their visit [3,4].

Like the concerns surrounding AI’s safeguards and transparency, measures for building trust in telemedicine were important considerations for using telemedicine platforms [20]. Finally, patients emphasized the importance of dermatologists addressing accessibility barriers, including the patient’s ability to navigate technology for telemedicine appointments [3,4], to ensure equitable care across diverse populations. This highlights important implications for health equity as teledermatology continues to expand.

Collectively, these findings suggest that patients may embrace AI and teledermatology only with appropriate safeguards, such as transparency about technological limitations, adequate educational resources, clear privacy policies, and, most importantly, continued dermatologist involvement that preserves the human elements of care. Figure 1 illustrates the interconnected drivers of patient receptivity, perceived benefits, and risks and provides a framework for integrated implementation strategies that address patient concerns while leveraging the complementary strengths of both technologies.

## Discussion

### Principal Findings

This narrative review of 48 studies revealed that patients exhibit distinct perspectives on AI and telemedicine in dermatology, with both technologies showing complementary strengths that could enhance dermatologic care delivery. While both technologies reduce wait times, they achieve this through different mechanisms—AI uses automated diagnostics and data analysis [1,28], whereas telemedicine minimizes logistical barriers, such as travel and appointment times [2,3,13,18,29]. This distinction is significant because it suggests implementation strategies should consider each technology’s unique advantages rather than applying a one-size-fits-all approach.

In addition, the sources of trust differ notably between the technologies. AI trustworthiness depends heavily on professional oversight, as patients strongly prefer AI that includes a dermatologist [28,43], and patients require AI models to be more accurate than dermatologists before they would feel comfortable with AI-only evaluations [43]. In contrast, telemedicine’s trustworthiness stems from patients’ confidence in its diagnostic capabilities for specific conditions, with studies showing high acceptance rates for certain dermatologic issues such as acne and infectious manifestations [17,22,38], while patients consistently preferred in-person evaluations for potentially malignant lesions [17]. Overall, patient acceptance of both technologies depends on perceptions of convenience, accessibility, and care quality, but concerns about privacy, data security, and remote consultation efficacy can impede implementation.

Our review also demonstrates how behavioral and contextual factors play a pivotal role in shaping patient receptivity to these technologies. Patterns of receptivity to AI and telemedicine appear closely tied to patient self-efficacy and contextual factors such as disease type, previous technology exposure, and institutional trust. For instance, patients managing chronic but nonsevere conditions such as acne or psoriasis often reported high satisfaction with teledermatology [18,19,38], which may reflect their familiarity with self-management practices. In contrast, patients with suspected malignancies or limited technology access expressed greater reluctance [15,17,35], likely reflecting both the high-stakes nature of cancer diagnosis that demands maximum clinical certainty and the digital barriers that prevent confident engagement with remote platforms.

These acceptance patterns are further complicated by the intersection of disease severity with demographic factors such as age and gender. Certain dermatologic conditions are more prevalent within specific demographic groups, such as acne more frequently and severely affects adolescent males [38]. These patterns raise the possibility that differences in technology acceptance based on disease severity may be confounded by underlying demographic factors. However, our review highlights the complex relationship between age and willingness to adopt the implementation of AI and telemedicine into dermatologic practice [14,17,30,36,43]. While younger patients who have more familiarity with technology may be more accepting of AI and telemedicine, they may also exhibit more hesitancy due to data privacy concerns [1,16,23,34,39,40]. This ambiguity indicates that technology acceptance is multifaceted and reflects the interplay between numerous clinical and demographic factors.

As the technological revolution expands, the convergence of AI and telemedicine in health care may become inevitable. These technologies may no longer remain distinct but instead function in tandem to provide a more integrated approach to care. Our results support that this integration should be gradual, and that AI and telemedicine should not replace traditional face-to-face services, but rather complement them [1,17,18,28,43].

While this simultaneous integration may exacerbate shared concerns—such as data privacy, loss of human interaction, and diagnostic accuracy—it also offers avenues to mitigate the risks inherent to each technology. For instance, AI tools could address concerns regarding the quality of teledermatology by standardizing image quality assessment and supporting diagnostic accuracy [8,9], while teledermatology’s real-time communication capabilities could mitigate patient concerns about AI’s lack of human interaction [1]. This synergy has the potential to enhance diagnostic accuracy, optimize resource allocation, and prioritize a patient-centered approach that maintains both technological efficiency and essential human touchpoints in health care delivery. As shown in Figure 1, successful integration requires addressing the overlapping concerns while capitalizing on each technology’s unique strengths through targeted implementation strategies.

It should be noted that, as dermatology care increasingly incorporates AI and telemedicine, attention to digital equity is paramount to prevent the widening of existing disparities. Several studies identified disparities in access, literacy, and comfort across demographic groups, and these findings foreshadow the implementation challenges discussed in the 4 paragraphs below, where the very populations that could benefit most from technological access may face the greatest barriers to adoption.

As our results outline, equity concerns surrounding AI center on algorithmic bias and representation, as evidenced by patients with darker Fitzpatrick skin types showing significantly lower acceptance of AI tools [26]. Meanwhile, equity concerns with teledermatology primarily relate to geographic and digital literacy disparities. Generally, patients with longer travel distances to the clinic are more accepting of telemedicine services, though for some patients, this preference may reflect travel or financial barriers rather than true choice [14,40]. Patients living in rural areas may particularly benefit from the increased convenience of web-, photo-, and app-based dermatology appointments but are simultaneously at a heightened risk of experiencing digital literacy and technology-related challenges [3,5,32,34,35].

Across both technologies, the introduction of new digital platforms without adequate support may disproportionately disadvantage individuals with limited digital literacy, including older adults, lower-income populations, and patients with lower education levels [14,17,30,35,36,39,43]. Without deliberate attention to inclusivity, the integration of these technologies risks reinforcing, rather than reducing, gaps in care access and quality. To address these equity concerns and increase patient acceptance across both technologies, implementation strategies must prioritize representative AI training datasets, intuitive telemedicine interfaces, transparent communication about both technologies’ capabilities and limitations, and patient-centered education.

Our review highlights that patients view successful integration of both technologies as requiring dermatologist oversight and the preservation of meaningful patient-provider relationships [3,43]. Therefore, offering ongoing resources and support throughout the integration process may help address patient concerns and maximize comfort with the platforms. Once these technologies are implemented, clinicians can begin by offering patients resources on how to use the AI or telemedicine service during in-person consultations, explaining how the technology works and emphasizing its role as a complementary tool that augments traditional care models [28,47]. Practices could also connect patients with follow-up resources, such as public libraries or help desks, to help train them on basic digital skills for health-oriented patient technology and empower them to engage fully with the services.

Beyond technical training, building trust necessitates transparent communication about security protocols and the protection of personal health information [23,46]. As the literature explains, endorsement from dermatologists alongside these trust-building measures may enhance patient acceptance of these technologies [39]. Ultimately, the success of these technologies in dermatologic care depends on thoughtful implementation that balances technological advancement with patient-centered care delivery. By ensuring that integration strategies align with patient concerns and expectations, these innovations can maximize their potential to improve access, efficiency, and quality in dermatologic care. The integrated framework shown in Figure 1 offers a roadmap for achieving this patient-centered technological convergence.

Although previous research has examined AI and telemedicine as separate modalities, little is known about how patients perceive their integration. Our search identified only 2 studies that addressed both technologies, and neither evaluated patient perspectives on their combined use. This likely reflects the novelty of such tools and the limited availability of integrated, patient-facing deployments during the review period. As these technologies evolve, future research focusing on patient trust and comfort with AI-augmented teledermatology will be important for guiding patient-centered implementation.

### Conclusion

In conclusion, patient perspectives surrounding AI and telemedicine in dermatology provide central considerations for clinical implementation. While patients value the benefits of improved access to care and reduced wait times, they continue to have concerns about data privacy, diagnostic accuracy, and maintaining meaningful doctor-patient relationships. These perspectives are especially important for informing health care accessibility in dermatology. As AI and telemedicine potentially converge in dermatologic care, balancing technological advancements with patient-centered care delivery should drive responsible implementation strategies. Research examining how patients experience these modalities together could guide health care systems in harnessing their complementary strengths, while continued investigation will be essential to understand how these technologies can best address patient needs in dermatologic care, both independently and in combination.

## Abbreviations

AI: artificial intelligence
PRISMA: Preferred Reporting Items for Systematic Reviews and Meta-Analyses
